# Post graduate clinical placements: evaluating benefits and challenges with a mixed methods cross sectional design

**DOI:** 10.1186/s12909-016-0575-7

**Published:** 2016-02-16

**Authors:** Jenny Yiend, Derek K. Tracy, Brian Sreenan, Valentina Cardi, Tina Foulkes, Katerina Koutsantoni, Eugenia Kravariti, Kate Tchanturia, Lucy Willmott, Sukhi Shergill, Gabriel Reedy

**Affiliations:** Department of Psychosis Studies, King’s College London, Denmark Hill, London, UK; Education Support Team, King’s College London, Denmark Hill, London, UK; Department of Psychological Medicine, King’s College London, Denmark Hill, London, UK; Oxleas NHS Foundation Trust, London, UK; Institute of Criminology, University of Cambridge, London, UK; King’s Learning Institute, King’s College London, Waterloo, London, UK

**Keywords:** Clinical placements, Postgraduate clinical education, Academic psychology

## Abstract

**Background:**

Systematic evaluations of clinical placements are rare, especially when offered alongside academic postgraduate courses. An evidence-based approach is important to allow pedagogically-driven provision, rather than that solely governed by opinion or market demand. Our evaluation assessed a voluntary clinical placement scheme allied to a mental health course.

**Methods:**

Data were collected over academic years 2010/11– 2013/14, from participating students (*n* = 20 to 58) and clinician supervisors (*n* = 10–12), using a mixed-methods cross-sectional design. Quantitative evaluation captured information on uptake, dropout, resource use, attitudes and experience, using standardized (the Placement Evaluation Questionnaire; the Scale To Assess the Therapeutic Relationship – Clinical version and the University of Toronto Placement Supervisor Evaluation) and bespoke questionnaires and audit data. Qualitative evaluation comprised two focus groups (5 clinicians, 5 students), to investigate attitudes, experience, perceived benefits, disadvantages and desired future developments. Data were analysed using framework analysis to identify *a priori* and emergent themes.

**Results:**

High uptake (around 70 placements per annum), low dropout (2–3 students per annum; 5 %) and positive focus group comments suggested placements successfully provided added value and catered sufficiently to student demand. Students’ responses confirmed that placements met expectations and the perception of benefit remained after completion with 70 % (*n* = 14) reporting an overall positive experience, 75 % (*n* = 15) reporting a pleasant learning experience, 60 % (*n* = 12) feeling that their clinical skills were enhanced and 85 % (*n* = 17) believing that it would benefit other students. Placements contributed the equivalent of seven full time unskilled posts per annum to local health care services. While qualitative data revealed perceived ‘mutual benefit’ for both students and clinicians, this was qualified by the inherent limitations of students’ time and expertise. Areas for development included fostering learning around professionalism and students’ confidence on placement.

**Conclusions:**

The addition of healthcare placements to academic postgraduate taught courses can improve their attractiveness to applicants, benefit healthcare services and enhance students’ perception of their learning experiences. Well-positioned and supported placement learning opportunities could become a key differentiator for academic courses, over potential competitors. However, the actual implications for student employability and achievement remain to be established.

## Background

Postgraduate academic courses offered in the health disciplines frequently provide practice placements, either as optional or compulsory components. Where placements are compulsory they are usually related to core clinical competencies required for a practitioner-oriented qualification and are subject to similar assessment requirements as the academic or theory-based course components. However, where placements are optional, they are usually offered as ‘added value’ elements of a primarily academic course. In this paper we focus on the latter and use the term ‘clinical placement’ to denote students attendance at weekly sessions in local healthcare service settings involving observation of, and contribution to, the routine activities of qualified clinical staff in providing mental healthcare to their patients.

We first consider what the drivers have been for academic courses’ increasing tendency, over recent decades, to offer such opportunities alongside their core academic teaching. We then go on to describe and evaluate one example of this model, implemented alongside an academic mental health course. The purpose of our study was to demonstrate a methodology and communicate findings that could serve as a model for other courses wishing to supplement compulsory academic content with ‘added-value’ optional clinical opportunities and to provide some examples of how such enhancements can be usefully evaluated. We finish by concluding on the concrete benefits such opportunities offer, and identifying areas for future research, to extend the current evaluation.

Numerous interacting factors have contributed to the increasing provision of voluntary, vocationally-based placements offered on postgraduate health professions courses. Perhaps the most obvious, to those familiar with the postgraduate higher education sector in the UK, are financial and political pressures. Government and higher education funding bodies no longer have the remit, or resources, to support academic pursuit as an end in itself at postgraduate level. Those days have largely passed following the gradual withdrawal of governmental support to institutions, and individuals, wishing to pursue higher education [1, 2]. Instead, there is an increasing political recognition that academic courses need to focus their provision towards giving graduates a competitive edge in their future workplace. One good example is the recent Higher Education Funding Council initiative to support Masters’ courses to develop models focused on ‘access to the professions’ [3, 4]. In subjects allied to health, this points to the addition to core provision of some form of clinical experience, training, or internship.

Another critical driver of the increasing placement provision offered by academic courses has been student demand. Students are well aware of the national consequences of wider factors such as the recent global recession, and fear for the impact of this on their own future prospects. By the time of reaching postgraduate study students already have concrete examples of friends, peers, and sometimes their own experience of competing in the graduate workforce marketplace. If the graduate job market is strained, then postgraduate study can be an attractive option for buying time to allow the market to improve, as well as enhancing one’s own employability in the meantime. Postgraduate students in the UK are now required to pay full tuition fees and therefore, at least in part, are acting as consumers in a higher education ‘marketplace’—indeed, that was the intention of UK government policy [5]. Student feedback is increasingly important and student satisfaction has become both an internal and external marker of course quality. Universities, and courses, are therefore responding to students’ expressed desire to incorporate professional experience into their courses, and to receive opportunities to develop and enhance their Curricula Vitae in a tightening employment market. The onus is therefore falling on Universities to demonstrate both value for money and enhanced employment opportunities for their post graduate students, and many are doing this using voluntary workplace placements and internships.

There is also a pedagogical driver to the increasing provision of placements. There is already evidence of the learning benefits which can accrue from the opportunity to develop theory-practice links [6], which offers prospects of enhanced learning experience in both practical and academic domains through a symbiotic relationship between the two. In addition, there is an inherent desirability to having complimentary practical experiences that can supplement core academic learning. For many, there is no substitute for hands-on experience in the domain of relevant clinical practice to truly confirm their motivation and ability to pursue the intended professional career; or, and equally usefully, to provide an early insight that alternative career pathways may be more suitable. This applies especially to careers in clinical psychology, where placements such as those evaluated in this paper, often provide the first real exposure to the clinical professional reality (e.g. contact with patients, clinicians and the National Health Service environment) of students’ desired career trajectories.

As a result of all these factors - political and financial pressures, student choice, enhancement of employment opportunities and transferable skills, pedagogical drivers - supplementary placement options are now seen as valuable elements by both the academic institutions offering them and students alike. However, systematic evaluations of their development, delivery and the concrete benefits they offer are, to date, rare. A review of the literature demonstrates that while there is considerable pedagogical research around professional healthcare placements, this has been largely restricted to those situations where clinical competency is a core element of the qualification itself. Indeed, this has led to helpful consensus on the kinds of attributes required of both clinicians and students, to foster the best possible learning experience [7, 8].

There is no guarantee that these same conclusions would apply in the somewhat different context of the optional clinical ‘experience’ placement that supplements an academic course. The lack of research on ‘added value’ clinically focussed additions to academic courses is perhaps surprising given their ascendance, as outlined previously. As educators, we have a duty to generate evidence of good practice by understanding, assessing and improving the benefits such developments provide. Doing so will allow academic courses to tailor the packages they offer at postgraduate level to actual, rather than perceived, needs and benefits of the student body they cater for and to ensure these are delivered going forward. Research on placement attachments can also help to align the learning objectives of all parties involved, which can otherwise differ in surprising ways [8]. Taking an informed, evidence-based approach allows the provision of optional professional placements on academic courses to become a pedagogically-driven, scholarly endeavour, rather than being governed solely by opinion and market demand [9]. Such an approach might seek to focus on professional standards and future accreditation needs, capturing how professional competencies focus on a combination of knowledge alongside the demonstration of clinical skills, which can only be achieved by in vivo exposure to practice settings [9, 10].

Against this background, the present paper reports an evaluation of the evolution of clinical placements as an optional addition to an academic postgraduate course, focusing on challenges and benefits for both students and clinicians. The aim of the current evaluation was to assess to what extent the clinical placement scheme met its objectives, which were are follows:

- to provide an added value experiential element to a primarily academic course
- to cater to student demand for clinical experience, as a means to enhance perceived future career prospects
- to increase students’ knowledge of the clinical workplace and offer them an opportunity to build relevant transferable skills and confidence
- to create a symbiotic relationship between future (students) and current (clinicians) mental healthcare service providers, resulting in mutual benefit

Data were collected over several academic years (2010/11–2013/14) from students and clinician supervisors who chose to participate in the course’s optional clinical placement scheme. The scheme ran alongside the compulsory academic modules of a one year full-time/two year part-time level 7 Master’s (QAA UK, 2014) in Mental Health Studies. The course typically attracts psychology graduates and those from allied disciplines (for example, biomedical sciences, neuroscience, social work, nursing, teaching and medicine) who are seeking to embark on a career (or enhance an existing pathway) as a qualified professional clinician (e.g., clinical psychology, graduate entry medicine).

## Methods

Sample sizes varied according to the measure and method of data collection used and are therefore reported individually in corresponding sections of the results. Ethical approval was granted for all aspects of the study by the London City Road and Hampstead Ethics Committee, reference 11/LO/1044. This included approval of written informed consent procedures which were administered to participants.

### Quantitative

We used quantitative evaluation to capture statistics on uptake, dropout, resource use and attitudes and experience.

#### Uptake and dropout

Projected student uptake was measured using an ‘opt-in’ postal survey emailed to all students ahead of the course start date, asking them to indicate whether they intended to take part in the course’s optional voluntary placement scheme. These figures were compared to actual uptake at the end of each year, as well as to data on those students dropping out after starting a placement.

#### Resource use

Use of clinical resources was captured by annual count of both the number of placements available and of the number of individual clinicians involved in placement supervision. Students’ contribution to resources was measured from the number of placements completed, totalling the hours of service per placement and calculating the full time equivalent contribution to clinical services.

#### Attitudes and experience

This was assessed by questionnaire and was available for specific cohorts only. Student attitudes at enrolment were assessed using a bespoke survey comprising 3 questions, each rated on an anchored Likert scale. Questions were: ‘How important was the placements scheme for you in choosing this course?’; ‘It is important for me to have clinical experience before finishing my Masters’ (both rated from 1 - not important at all, to 10 - very important); and ‘The Clinical Placements Scheme was one of the main reasons I applied for Mental Health Studies over other courses’ (rated from 1- strongly disagree, to 5- strongly agree).

We conducted a comprehensive search of the literature to identify the most suitable published questionnaire measures to assess students’ and clinicians’ post placement attitudes and experience. Only three instruments with direct relevance to placements were found: the Placement Evaluation Questionnaire (PEQ; [11]); the Scale to Assess the Therapeutic Relationship (STAR-C; [12]) and the University of Toronto Placement Supervisor Evaluation (PSE; [13]).

The PEQ ([11]) is a 12-item Likert-style questionnaire, which has been used in previous studies to obtain feedback from students in clinical learning environments [11] and was used here. It covers overall experience, preparation/ induction, the learning experience, skills acquisition, support and confidence. Participants rate each statement from 1 - strongly disagree, through 3 - neither agree nor disagree, to 5 - strongly agree. There are no reverse-scored items.

The STAR [12]) is a brief instrument assessing the perceived quality of the therapeutic relationship in psychiatric settings and comprises 12 Likert-style questions. Participants rate each statement from 1 - strongly disagree, through 3 - neither agree nor disagree, to 5 - strongly agree. Logistical reasons prevented using the patient version, but the students completed the clinician version (STAR-C). It was used to gauge the degree to which students had felt able to, or had the opportunity to, establish beneficial therapeutic relationships with patients while on placement.

Supervisors’ attitudes and experience of placement provision was assessed using the University of Toronto Placement Supervisor Evaluation (PSE; [13]). This is an 18-item Likert scale questionnaire that enquires about student progress under four broad categories: Learning Objectives (items 1,2), Attitude (items 3–8), Performance (9–14), and Communication (15–18).

### Qualitative

We used qualitative evaluation to provide a deeper understanding of students’ and supervisors’ attitudes and experience of the scheme, including perceived benefits, disadvantages and desired future developments. Focus groups were chosen to enable flexibility, stimulate discussion and readily observe and explore any similarity or divergence of viewpoint [14].

A student focus group was held with five randomly selected students who had participated in the placement scheme. A topic guide was agreed, covering three main themes: experience of placement; benefits of placement; and career path. Core questions included: ‘What did you learn?’; ‘How did you find the patients?’; ‘How has the placement helped you decide what you want to do?’ and ‘What were the benefits of the placement?’ The focus group was audio recorded and subsequently transcribed with the data anonymised.

A clinician focus group was held with five volunteer clinicians who had participated as supervisors, again covering the themes: experience of placement and benefits of placement; as well as, clinician recruitment. Core questions included: ‘What did you learn?’; ‘How did you find the students?’; ‘What are the barriers to clinicians getting involved?’ and ‘What were the benefits of the placement?’ The focus group was recorded, transcribed and data anonymised.

The data were analysed using framework analysis [15, 16] to identify *a priori* and emergent themes. The use of framework analysis was decided upon in consultation with colleagues, and in conjunction with an experienced qualitative researcher (LW). Srivastava & Thomson argue [16] that this qualitative method is well-adapted to research that has specific questions, a limited time frame, a pre-determined sample and *a priori* areas of interest. According to Ritchie & Spencer [15], framework analysis may generate theories; however, the main concern is to describe and interpret what is happening in a particular setting. In the analysis stage the gathered data were sifted, charted and sorted in accordance with key identified issues and themes. This involved a five-step process: 1. familiarization; 2. identifying a thematic framework; 3. indexing; 4. charting; and 5. mapping and interpretation [15]. Three members of the team (LW, JY, BS) were involved in running the focus groups and coding and categorising the data through a process of consensus discussion. LW was independent from all aspects of the course under evaluation and this input served as a check on any researcher bias.

## Results

### Quantitative

#### Uptake

An initial pilot phase in 2009/10 offered 12 trial placements to students, selected on the basis of their suitability to available placements. Thereafter the scheme progressively grew over the three subsequent years, as shown in Table 1. Uptake had stabilised by 2013/14 at around 70 placements per annum, catering for around three quarters of the cohort. These placements were typically provided by about 20 individual clinicians per annum, though considerably more (57 in 2013/14) made initial enquiries about the scheme each year (see Table 1, Uptake). This pattern, which was consistent over the 3 cohorts (Table 1), indicated that the majority of initial contacts made by potentially interested clinicians did not translate into concrete placement opportunities; furthermore, many supervising clinicians took on several students per annum.

**Table 1:** Uptake, dropout and resource contribution by academic year group

	Uptake				Resource use and contribution			Dropout
Academic Cohort	Total eligible students, *n*	Initial student interest, *n* (%)	Clinical contacts approached	Clinicians offering placements, *n* (%)	Total placements offered, *n* (%)	Total hours service	Full time equivalent working contribution^a^	Placements completed, *n* (%)
2010/11	106	48 (45)	45	12 (27)	33 (31)	2529	1.4	32 (97)
2011/12	110	61 (55)	71	24 (34)	50 (45)	6014	3.3	48 (96)
2012/13	107	74 (69)	53	20 (38)	70 (65)	-	-	-
2013/14	106	68 (64)	50	27 (54)	71 (67)	12921	7.2	53 (96)

^a^assuming one full time post comprises 1800 h per annum (37.5 h per week, for 48 weeks)

#### Drop out

Drop out was very low across all years, never rising above 5 % (typically 2–3 students). This matches average annual course dropout/ deferral rates. Investigation of individual cases showed that reasons for dropout were related to factors not specific to placements themselves, but rather affecting the students’ engagement in the course overall (e.g. personal circumstances requiring interruption from studies or withdrawal from the course).

#### Resource use

Students were limited to a maximum of one day per week on placement (due to other course commitments), within which they had to fit their induction, training and general familiarisation with the service. Nevertheless, analysis of resource use showed that students on the scheme contributed the equivalent of 3.3 full time equivalent posts (FTE) to local mental healthcare services in 2011/12, rising to around 7 FTE in 2012/13 and 2013/14.

#### Attitudes and experience

Questionnaire data from students, provided in Table 2, revealed that 62 % (2012/13 cohort) indicated that the scheme was one of the main drivers behind their choice of course. Post placement evaluation questionnaires showed a largely positive experience for both placement supervisors and students. For students completing the placement evaluation questionnaire, 70 % (*n* = 14) rated it as an overall positive experience, 75 % (*n* = 15) reported that the placement was a pleasant learning experience, 60 % (*n* = 12) stated that it enhanced their clinical skills and 85 % (*n* = 17) believed that it would benefit other students. The STAR, assessing the therapeutic relationship between student and patients, was completed by 63 % of the sample. Individual responses are shown in Table 1, but this measure is also designed for computation of a mean overall score for which normative data is available [12]. Our student sample mean was 32.2 (SD = 6), which is similar to that reported for trained clinical therapists and their patients, at 31.5 (SD = 7).

**Table 2:** Questionnaire data on student attitudes and experience

Attitudes at enrolment				Placement evaluation questionnaire				Scale to assess therapeutic relationship			
2012/13 cohort, *N* = 58				2010/11 cohort, *N* = 20 (63 % of completers)				2010/11 cohort, *N* = 20 (63 % of completers)			
	Not important (1–3)	Somewhat (4–6)	Important (7–10)		Disagree (1–2)	Neutral (3)	Agree (4–5)		Disagree (1–2)	Neutral (3)	Agree (4–5)
Placement important in course choice	13 (22 %)	9 (15 %)	36 (62 %)	Pleasant learning experience	2	3	15	We got along well	1	1	18
Clinical experience before finishing	3 (5 %)	8 (14 %)	47 (81 %)	I felt well prepared	2	6	12	We shared good rapport	1	1	18
	Disagree (1–2)	Neutral (3)	Agree (4–5)	Met my objectives	2	5	13	I listened to patients	1	0	19
Placements as reason for course choice	15 (26 %)	7 (12 %)	36 (62 %)	Placement assisted learning	2	6	12	Patient rejected me	16	2	2
				Enhanced clinical skills	5	3	12	Shared a good relationship	1	1	18
				Supported professional growth	2	3	15	I felt inferior to patient	16	2	2
				Adequate instruction	4	4	12	We shared similar expectations	2	13	5
				Expected at venue	1	6	13	I was supportive of my patient	0	2	18
				Staff willing to assist	2	4	14	Difficult to empathize	14	4	2
				Feel confident working there	1	5	14	We were open with each other	0	10	10
				Many learning opportunities	3	4	13	Could take patient’s perspective	1	3	16
				Experience would benefit others	1	2	17	We shared trusting relationship	1	8	11

**Table. 3:** Questionnaire data on clinician attitudes and experience: University of Toronto placement supervisor evaluation

	2011/12 academic year, *N* = 10 (42 % of clinicians)			2013/14 academic year, *N* = 12 (43 % of clinicians)		
	Disagree	Neutral	Agree	Disagree	Neutral	Agree
	(1–2)	−3	(4–5)	(1–2)	−3	(4–5)
1. Student could apply academic concepts/approaches to service activity	1	0	9	1	4	7
2. Student demonstrated recognition of and appreciation for the unique knowledge and/or skills possessed by those s/he worked with	0	0	10	0	2	10
3. Student exhibited enthusiasm for service activities (*positive attitude*)	0	0	10	0	3	9
4. Student demonstrated sensitivity toward the people with whom s/he worked	0	0	10	0	1	11
5. Student dealt positively with uncertainty and setbacks (*adaptable*)	0	0	10	1	2	9
6. Student exhibited a sincere desire to learn	0	0	10	0	1	11
7. Student appeared motivated	0	0	10	0	3	9
*8.* Student exhibited initiative *(asked questions, scheduled meetings, came up with ideas, etc.)*	1	1	8	0	5	7
*9.* Student demonstrated responsibility *(time management, attendance, punctuality, reliability, etc.)*	1	1	8	0	1	11
*10.* Student was able to work independently *(accountable, self-directed, etc.)*	1	1	8	0	2	10
11. Student demonstrated commitment (*completion of tasks, consistency, etc*.)	1	1	8	0	3	9
*12.* Student experienced a growth in understanding *(depth of awareness, appreciation for complexity, etc.)*	0	2	8	0	1	11
*13.* Student exhibited decision making and problem solving skills *(recognizing and evaluating options, executing a plan of action, etc.)*	2	3	5	1	2	9
14. Overall, student made a significant contribution to the service.	3	3	4	0	1	11
15. Student responded thoughtfully to critical feedback and suggestions	0	1	9	0	0	12
16. Student exhibited professionalism (*maturity, respect, confidentiality, etc.*)	0	1	9	0	1	11
17. Student developed an understanding of and competency in the unique styles of communication used in the placement context	0	2	8	0	2	10
*18.* Student worked well with individuals from different backgrounds, interests and experiences *(including peers, supervisors and community services)*	0	3	7	0	1	11

Placement providers also reported largely positive experiences as shown in Table 3. Forty-two per cent (*n* = 10) completed the University of Toronto Placement Evaluation Survey, with most (*n* = 9) reporting that students had met their learning objectives and exhibited good communication skills. All considered that students demonstrated good appreciation of the knowledge and skills of those they worked with and showed respect and eagerness to learn. Overall, student performance was rated “good” by 60 % (*n* = 6) and all providers found students to have a good attitude towards their duties. Fewer, 40 % (*n* = 4), agreed that students made a significant contribution towards the service, possibly reflecting the limits imposed by students being clinically unqualified and having limited time available (a maximum of one day per week) at the individual level.

### Qualitative

**Fig. 1:**
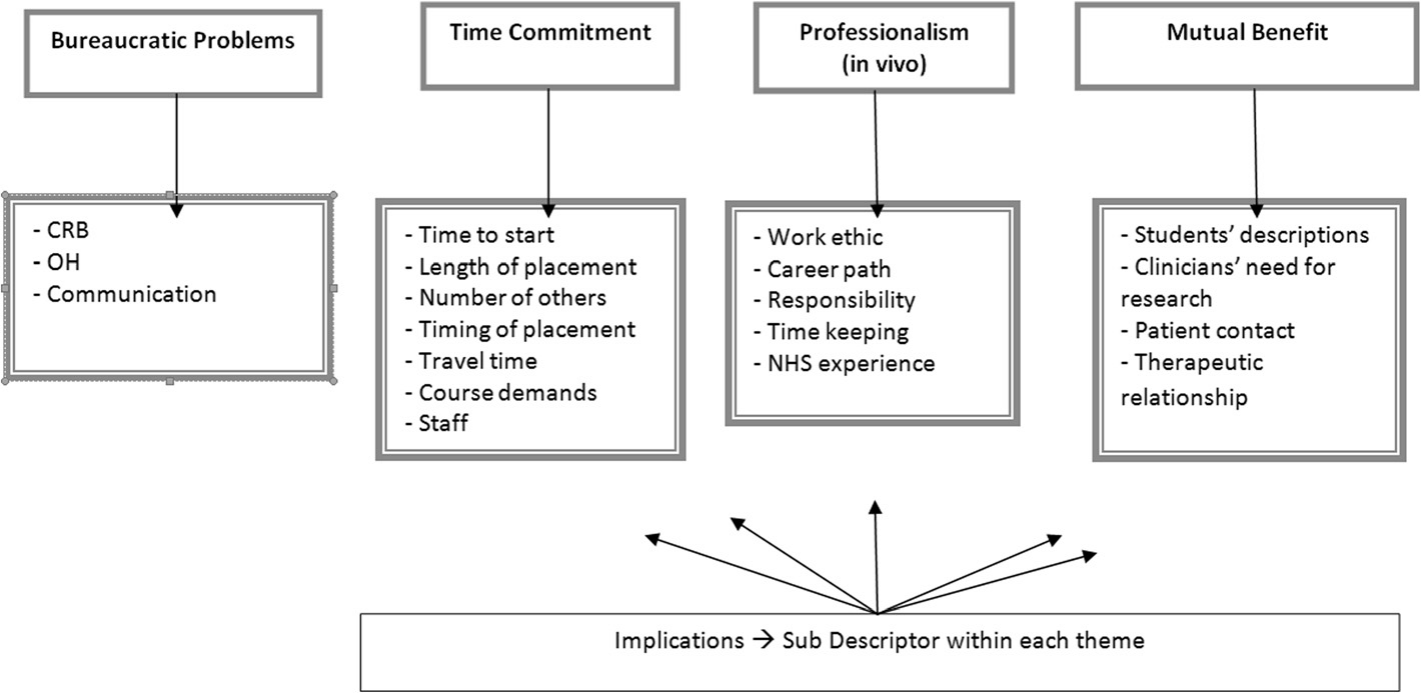
Themes and subdescriptors from qualitative analysis. Detailed legend: The figure shows the results of the qualitative analysis of transcripts from each focus group, one with students and one with supervisors. The same themes emerged from each and thus are illustrated together in the figure. Broad themes (Mutual Benefit; Professionalism; Time Commitment and Bureaucratic Problems) subsumed more detailed subdescriptors within each theme. CRB: Criminal Records Bureau. These are compulsory statutory checks administered by the UK government which identify any previous criminal convictions of a potential employee which might render them unsuitable for work in the proposed position. OH: Occupational Health. These are compulsory institutional checks to identify any health issues which need special provision or which might render a potential employee unsuitable for work in the proposed position

While results were initially analysed separately for each focus group (students, supervisors), it became clear that the same themes were emerging, thus data were analysed and are reported together. Analysis highlighted the following themes: Mutual Benefit; Professionalism; Time Commitment and Bureaucratic Problems, as shown in Fig. 1. Sub descriptors within each theme are also listed in Fig. 1.

#### Mutual benefit

This theme reflected the benefits of participation in placements for the service, the clinicians and the students. Benefits to the service included the new resource provided (as also identified in the quantitative data). Benefits to the students included their perceptions of the added value their clinical experiences were providing to their academic qualification. Clinicians reported significant benefits from hosting placements, such as fostering local research and students’ offering a fresh perspective on clinical situations. Students welcomed the chance to observe and, in some cases, practise clinical activities, and felt a sense of their own contribution.

*‘I can volunteer and help out with bits …because they have 1 million other things to do..*.’ *(Student 1)*

*‘they can bring things they’ve learned and apply it’ (Clinician 3)*

#### Professionalism

This theme reflected a learning need around work ethic and professional values and practices, highlighting that this is not usually taught in academic courses but is essential to clinical working life. Professional attitudes were stated as important learning outcomes of the placements by both groups. Clinicians noticed differences between individual students in work ethic and basic professional skills. Students noticed the benefits of gaining an increased understanding of professionalism, and were able to practise acquisition of those skills, which included confidence, responsibility and integration.

*'I would be much more suited to a job in that area now' (Student 3)*

*‘We’re here …to model professional behaviour’ (Clinician 2).*

#### Time commitment

Time commitments were mentioned by all participants as an important factor in clinical placements. There was different emphasis placed on the needs of the student compared to those of the placement. Students required flexibility in their placement to enable them to maximise academic and practical learning in accordance with course and personal priorities. Clinicians identified the initial and ongoing time commitment of induction and supervision but recognised this could be offset through the extra resource of a well-placed, motivated student. Students did not feel obliged to continue working in placements at exam time and clinicians focused their consideration on how to best utilise the student as a resource.

*‘during exams … I didn’t necessarily have time to see the patients…they [placement provider] were really helpful with taking some [patients] off my hands if I was having trouble…’ (Student 3)*

*‘I clearly reduced numbers [of students on placement] and it helped’ (Clinician 1)*

#### Bureaucratic problems

Bureaucratic problems were mentioned by all but one student and by all clinicians. Both sides identified a number of bureaucratic barriers to their placements. The students commented on the Criminal Records (CR) clearance procedures and Occupational Health (OH) clearances. Some students found the OH process quite an ordeal, whereas the CR process was seen to be much more manageable, although still onerous. Likewise, clinicians stated that these processes and their own local approvals were time consuming and labour intensive. In some cases these procedures prevented the timely uptake of available placements. Clinicians commented on the communication processes, suggesting a more streamlined approach whereby dedicated administrative support might serve to increase efficiency levels on all sides.

*‘They wanted the actual clearance and the clearance letter ((OH) Occupational Health) you need to get. It was a bit painful’ (Student 1)*

*‘CR checks etc., you know this creates quite a lot of work’ (Clinician 1)*

## Discussion

The original aims of the scheme were largely met. Our first aim was to provide an added value experiential element to a primarily academic course. Relatively high uptake figures, low dropout and positive focus group comments provide evidence that this aim was achieved. The second aim was to cater to student demand for clinical experience providing the perceived enhancement of future career prospects. Figures across four academic cohorts showed that this aim was also met. Students’ own evaluation confirmed that placements met expectations and that their perception of benefit remained after completion. Our third aim was to increase students’ knowledge of the clinical workplace and offer them the opportunity to build relevant transferable skills and confidence. Data revealed that in some cases this was met, although confidence did not improve as much as expected. Finally, we set out to create a symbiotic relationship between students (would-be future healthcare providers) and current clinicians working in mental healthcare services. Data did indeed reveal a perceived ‘mutual benefit’ according to focus group evaluation; however, this was qualified by the inherent limitations of students’ time and expertise.

Quantitative results highlighted that uptake from individual clinical services was lower than initial expressions of interest suggested. Those who did engage had uniformly positive experiences, suggesting that further research to explore initial barriers to participation would be helpful. Student drop out was extremely low (3 students, 4 %, maximum), and was accounted for by wider contextual and personal factors, suggesting that for the majority initial enthusiasm was matched by ongoing commitment. Analysis of resources indicated that the scheme contributed significantly to local healthcare services, by providing the equivalent of seven full-time posts annually. A full time entry-level UK graduate position (e.g. a clinically unskilled, unqualified research assistant or support worker) currently costs the service £37,620 (including employer contributions and an urban ‘weighting’ payment). Seven FTE thus contributed £263,340 to local healthcare services. This, of course, represents additional manpower devoid of any clinical expertise or qualification. However national healthcare services typically employ around 47 % (547,000) non-clinically qualified staff (2013 data; Health and Social Care Information Centre, 2014). In addition this financial contribution does not take induction, training, supervision, infrastructural or administrative costs into account, which further limits the impact. It is nonetheless an important emergent finding from our study that placement students made a significant contribution to mental healthcare provision; a finding that may aid other courses in implementing similar schemes.

Questionnaire evaluation revealed that both students and clinicians had positive experiences of the scheme; however the low completion numbers for some cohorts mean that some caution must be attached to data interpretation. Of particular note, the majority of students (85 %) across all cohorts reported that it had enhanced their clinical skills and would benefit others, which is perhaps in part testament to the positive characteristics of those professionals with whom they were most closely working, as Buchel and Edwards [17] suggest. Furthermore, students perceived that they had built clinical therapeutic relationships with their patients while on placement, which were at least as good as those typically reported by practising clinicians, as measured by a standardised clinical instrument. This suggests that, in the students’ view at least, the placement scheme largely met its educational objectives of supplementing academic learning about mental health disorders by enhancing translational skills, professional knowledge and relevant experience. Likewise, clinical placement providers also reported positive experiences. Of note, all providers felt that students had demonstrated a good appreciation of the knowledge and skills of their professional colleagues, had shown respect and eagerness to learn, and most considered student attitudes and overall performance to be good. One interesting point of discrepancy, however, was that fewer than half of clinicians felt that students had made a significant contribution towards the service. Thus, while overall resource contribution to the scheme was significant, at the individual level this was not necessarily the perception of clinical placement supervisors. This is perhaps not surprising, given the limited time commitment and clinical expertise of the students.

Qualitative findings triangulated with, but also added to, the quantitative results summarised previously. The more in-depth analysis, which is characteristic of qualitative work, provided important additional insights, with implications for how the scheme could be further improved. The theme ‘Mutual Benefit’ reflected many of the positive quantitative findings summarised earlier, confirming the contribution of a new resource to services and students’ positive perceptions of the added value of their learning experiences while on placement. ‘Professionalism’ reflected the relative naivety of the student participants to the standards and norms prevalent in the healthcare workplace. While the scheme was clearly instrumental in teaching these values, the data highlighted a potential need for more structured teaching around this topic prior to commencing placement. Interestingly, this same theme has been highlighted as important by research in the medical domain [3]. While noting the importance of the concept of professionalism, particularly in the context of misconduct risk within professional practice, Tiffin and colleagues nevertheless found it to be a particularly difficult construct to measure using standardised tests, which suggests that softer approaches might be more suitable. On the other hand, Chipchase and colleagues [18] have conducted a useful consensus analysis of 258 clinicians’ perceptions of what makes a suitably well-prepared student, recommending six themes (57 individual characteristics), including Professionalism, which could be used to help prepare students for placements ahead of starting.

‘Time Commitment’ arose as a theme for students and clinicians, in the latter case indicating a possible reason why other clinicians may not be participating, despite initial interest. In over-stretched, under-resourced services (a common scenario in current UK healthcare provision), concerns around potential time consuming extra supervision load may have been overriding the perceived potential benefits of having an additional pair of hands. It is worth noting that this theme is likely to be less applicable in contexts where courses have compulsory rather than optional placements, because the time on placement will be seen by both parties as an inherent part of the course, rather than an optional extra, meaning that both student and provider have little cause to consider questions of additional or unnecessary time consumption.

Perhaps the most illuminating theme was that of ‘Bureaucratic Problems’, which was a key issue raised by students and clinicians alike. Complex and lengthy procedures for obtaining necessary approvals prior to patient contact were perceived by most as challenging and potentially prohibitive. An obvious implication of this is the need for additional administrative resource, provided by the course itself, to coordinate and streamline these procedures. Substantial additional administrative support is required if academic courses are to offer successful additions of practical healthcare learning opportunities to students.

One domain which did not figure prominently in our qualitative data was that of confidence. Given that for many students this would have been their first exposure to the realities of patient contact and clinical challenges, we were somewhat surprised at the absence of this theme. Indeed, other work has shown that confidence is a crucial skill for which practice placements can provide the ideal development opportunity: after all, they importantly provide the relatively simple experience of interaction with patients and clinicians and the development, through exposure, of maturity and a sense of personal agency [10]. It may be that the relatively high competition for places on the course evaluated here (around four applicants per place) acts as an inherent self-selection mechanism, yielding only those more confident students. Nevertheless, future work in this area might seek to investigate confidence as an *a priori* topic of interest.

This study had some notable strengths, but also a number of limitations. Using a mixed methods design was a clear strength, as we were able to combine hard factual data with more in depth analysis of attitudes and perceptions. This combination served to both triangulate the findings, as well as identify areas for improvement that would otherwise have been missed. A further strength was the ability to track the development and stabilisation of the scheme over a number of years, providing a useful longitudinal insight into the development and sustainability of the new initiative, although this was compromised somewhat by missing data for some cohorts.

An obvious limitation was that we were not able to include a hard outcome measure of impact of the addition of the new scheme, such as subsequent employability, or successful entry to professional training (such as clinical psychology programmes). It is notoriously difficult to track students’ progress upon leaving higher education, meaning that any obtainable figures are inevitably biased by self-selection of respondents. Another important outcome measure, which future work might seek to address, would be to include an assessment of the scheme’s impact on academic grades, to address the question of whether there might be a trade-off between practical experience and academic performance. It would presumably be important to students and lecturers alike to know that grades were not being compromised by the additional time and energy being devoted to non-assessed, optional placement activity. Additionally, it would be informative in future work to assess students’ (and course providers’) perceptions about whether, and how, their practical experiences on placement complemented didactic teaching on their course. One might hypothesize that the combination of academic learning with the opportunity to observe and apply this in practice might provide bidirectional added value to both components of the educational experience.

A further cautionary note is that the findings we present are, of course, limited in scope to the particular programme concerned. Further, the placements were voluntary, which may introduce an intrinsic sample bias in terms of the students choosing to undertake these placements, although the majority of the student population took part. Furthermore there were a limited number of placements and in some instances the sample size pertaining to the evaluation data is small. Nevertheless we argue that the considerations raised by this work may be useful in informing the discussions of other programmes that either currently feature, or plan to implement, similar added-value professional experience schemes.

Future work should consider a more in-depth analysis of the pedagogy behind the learning processes while on placement. As noted in the introduction, it is crucial that educational developments are driven by sound pedagogical principles and data. A good example of such an approach is that offered by Delany and Bragg [9], in which focus group work revealed some very different conceptions of the learning process between the clinical educators and their physiotherapy students on practice placements. Educators focussed on conveying structured knowledge in a series of discrete steps, while students - although appreciative of filling their knowledge gaps - conceived their learning as a more dynamic process of identifying contacts and techniques to develop their understanding. Similarly, academic and clinical educators do not always agree on the most important attributes to be developed during placement attachments [8]. Aligning the learning expectations and objectives of practice placements across students, academics and clinical supervisors - and making these explicit to all parties - will be an important agenda for the future of added value postgraduate components. Finally, future work might benefit by embedding within a socio-cultural theoretical framework such as the social learning system (SLS; [19]). A SLS is a social network containing ‘communities of practice’ (CoPs; [20]) and within which learning takes place. Learning arises, within CoPs, from interactions between individuals and their personal experiences when these are shared around a common passion, interest or goal. The medical education literature provides good examples of the use of the SLS framework to enhance our pedagogical understanding of the learning process during placement activities and these might serve as a template for future work within similar nonmedical settings ([21–23]).

## Conclusions

The addition of healthcare placements to academic postgraduate taught courses can improve desirability, benefit healthcare services and enhance students’ perception of their learning experiences. Well-positioned and supported placement learning opportunities can be a key differentiator for academic courses, over potential competitors. The methodology and findings presented here could be useful as a model for other courses wishing to supplement academic content with similar clinical experiential opportunities and provide some examples of how such enhancements can be usefully evaluated. However, the actual implications for student employability and achievement remain to be established.
